# Immunotoxicological Evaluation of Genetically Modified Rice Expressing Cry1Ab/Ac Protein (TT51-1) by a 6-Month Feeding Study on Cynomolgus Monkeys

**DOI:** 10.1371/journal.pone.0163879

**Published:** 2016-09-29

**Authors:** Xiaoyan Tan, Xiaobing Zhou, Yao Tang, Jianjun Lv, Lin Zhang, Li Sun, Yanwei Yang, Yufa Miao, Hua Jiang, Gaofeng Chen, Zhiying Huang, Xue Wang

**Affiliations:** 1 Key Laboratory of Beijing for Safety Evaluation of Drugs, National Center for Safety Evaluation of Drugs, National Institutes for Food and Drug Control, Beijing, People’s Republic of China; 2 Center of Safety Evaluation on New Drug, School of Pharmaceutical Science, Sun Yat-sen University, Guangzhou, Guangdong Province, People’s Republic of China; Huazhong University of Science and Technology, CHINA

## Abstract

The present study was performed to evaluate the food safety of TT51-1, a new type of genetically modified rice that expresses the Cry1Ab/Ac protein (Bt toxin) and is highly resistant to most lepidopteran pests. Sixteen male and 16 female cynomolgus monkeys were randomly divided into four groups: conventional rice (non-genetically modified rice, non-GM rice), positive control, 17.5% genetically modified rice (GM rice) and 70% GM rice. Monkeys in the non-GM rice, positive control, and GM rice groups were fed on diets containing 70% non-GM rice, 17.5% GM rice or 70% GM rice, respectively, for 182 days, whereas animals in the positive group were intravenously injected with cyclophosphamide every other day for a total of four injections before the last treatment. Six months of treatment did not yield abnormal observations. Specifically, the following parameters did not significantly differ between the non-GM rice group and GM rice groups: body weight, food consumption, electrocardiogram, hematology, immuno-phenotyping of lymphocytes in the peripheral blood, mitogen-induced peripheral blood lymphocyte proliferation, splenocyte proliferation, KLH-T cell-dependent antibody response, organ weights and ratios, and histological appearance (*p*>0.05). Animals from the GM rice group differed from animals in the non-GM rice group (*p*<0.05) in several parameters: specifically, their body temperatures and serum alanine aminotransferase (ALT) levels were higher, whereas their levels of serum K^+^, Cl^-^ and cytokines (IL-2, IL-4 and IL-5) were lower. Because dose- or time-dependent changes were not observed in this study and animals appeared histologically normal, the aforementioned differences were not considered to be adverse or related to the treatment with GM rice. In conclusion, a 6-month feeding study of TT51-1 did not show adverse immunotoxicological effects on cynomolgus monkeys.

## Introduction

*Oryza sativa L* is one of the most important food crops, as it provides more than 20 percent of the world’s energy and more than 15 percent of the total food protein supply for humans. In 2013, rice was planted on approximately 164 million hectares, 18 percent of which was planted in China [1]. Thus, improving rice yield is important for the Chinese economy. However, lepidopteran pests, such as stem borers and rice leaf folders, severely impact the harvest of rice, and lepidopteran-resistant plants are not available. Therefore, pesticides are frequently used to prevent pests, which leads to serious environmental pollution and increases the cost of rice production. Alternatively, genetic engineering strategies may be used to prevent rice pests and increase rice in a safe and environmentally friendly manner.

*Bacillus thuringiensis* (Bt) genes have been widely used to generate genetically modified (GM) crops because the expressed proteins confer specific resistance to lepidopteran pests. This strategy is the most cost-effective way to prevent pests. In recent decades, various types of genetically modified rice expressing Bt genes have been developed. HuaHui 1 (TT51-1) is a patented new type of GM Bt rice developed by the Central China Agricultural University that is currently being investigated in China. It was created by inserting a synthetic fusion gene of *cry1Ab/Ac* into the parental rice strain, Minghui63, via particle bombardment, and expresses the Cry1Ab/Ac protein (Bt toxin) [2]. TT51-1 has received two biosecurity certificates from China’s Ministry of Agriculture as a GM plant of safety level-II. Field tests indicate that TT51-1 is highly resistant to most lepidopteran pests–it exhibited full protection in the presence of many pests and absence of chemical insecticides.

Because the foreign genes in GM foods did not originate from traditional gene banks, the proteins expressed by these genes may lead to food safety problems such as allergic reactivity, and the safety of GM foods has attracted significant attention worldwide and has been the topic of heated discussion by the public. Thus, safety evaluations are necessary prior to commercialization to ensure the safety of GM foods. For TT51-1, the current food safety data are insufficient. Specifically, current studies did not identify differences in the main nutritional components between TT51-1 and its parental rice, acute and irreversible toxicities or teratogenic and carcinogenic toxicities due to TT51-1 were not observed in rats [3–5]. Therefore, this transgenic Bt rice was identified as a GM product of safety level-I, the highest safety level of GM products.

Previous studies showed that the NOEL (no observed effects level) of Bt products was higher than 8400 mg/kg [6], and that of Bt rice was higher than 20 g/kg, which classifies them as non-toxic substances [7]. Genetically modified rice containing Bt genes was not toxic to mice or rats after oral administration for 28 days or 90 days [8–11]. Furthermore, decreases in the adrenals weights and changes of in the clinical biochemistry, such as TP, CRE, CHOL, MCV, and HCT, were observed in rats treated with genetically modified rice containing Bt genes [12,13].

However, safety studies of GM Bt rice have primarily been conducted in rodents and consisted of 30-day or 90-day feeding studies, and safety data in primates are lacking. The effects of GM Bt rice on primates are unknown, especially the effects on the immune system. To enrich food safety data of GM Bt rice and provide more scientific data for food safety risk assessment and the development of technologies to evaluate the safety of GM crops, this study examined the potential influences of TT51-1 on the immune system of cynomolgus monkeys.

## Materials and Methods

### Test materials

The genetically modified Bt rice (GM rice, TT51-1) was developed by the Central China Agricultural University (Wuhan, China), and seeds of GM rice and its corresponding parental rice (MingHui63) were harvested in Jiangxi Province, whereas seeds of conventional rice (non-GM rice) were purchased from the market. The nutrient compositions of GM rice, parental rice and non-GM rice were determined in accordance with standard Chinese methods, as described by Wang et al[4]. The levels of Cry1Ab/Ac protein that accumulated in the rice seeds were measured using a specific enzyme-linked immunosorbent assay (ELISA) for Cry1Ab/Ac (Agdia, PSP 06200, USA). Monkeys were fed on diets containing either 70% GM rice or 70% non-GM rice (w/w). A third group of monkeys received a diet containing 17.5% GM rice and 52.5% parental rice (w/w), which constituted the low-dosage group. All diets were nutritionally balanced and met the nutrients requirements for monkeys (GB/T 14924.3–2010) (Table 1). The diets were manufactured and analyzed by Beijing Keaoxieli Feed Co., Ltd. (Beijing, China). Three random samples were selected from each diet for compositional analysis. The nutrients in these diets, i.e., the crude protein, crude fat, crude fiber, moisture, crude ash, calcium and phosphorus contents, were analyzed in accordance with standard Chinese methods for feedstuff analyses (GB/T6432-1994, GB/T6433-2006, GB/T6434-2002, GB/T6435–2006, GB/T6438-2007, GB/T6436-2002, and GB/T6437-2006).

**Table 1:** Nutrient composition of non-GM rice, GM rice and its parental rice.

Contents	Non-GM rice ^a^	GM rice	Parental rice ^b^
Bt protein (μg/g)	0.0	2.5	0.0
Crude protein (g/kg)	80.4	63.4	63.9
Crude fat (g/kg)	5.0	6.9	8.5
Crude fiber (g/kg)	4.7	8.3	7.7
Moisture (g/kg)	15.1	141.2	138.0
Crude ash (g/kg)	4.1	5.8	5.3
Calcium (g/kg)	0.3	0.2	0.4
Phosphorus (g/kg)	0.9	1.2	1.2

^a^: Non-GM rice was purchased from the market

^b^:The parental rice was MingHui 63.

### Animals

Sixteen male and sixteen female 2- to 3-year-old cynomolgus monkeys (*Macaca fascicularis*) were obtained from Guangxi Xiongsen Primate Experimental Animals co. Ltd. (Guigang, Guangxi Province, China). All animal experiments were conducted at the National Center for Safety Evaluation of Drugs (NCSED) of National Institutes for Food and Drug Control (Beijing, China) following approval by the Institutional Animal Care and Use Committee(IACUC) of NCSED (Approval NO: IACUC-2013-065). Monkeys were individually maintained in stainless steel cages (*L×W×H*: 800×700×750 mm) under condition of 16–26°C, 40–70% relative humidity, a 12-h light-dark cycle and a room air exchange of 8–10 times per hour. Each monkey was provided with 200 g of special monkey keeping diet and approximately 150 g of fruit approximately per day, and sterilized water was offered *ad libitum*. Each monkey was provided with toys (such as mirror) and opportunities for communications with the neighbors, and was monitored daily by animal care staff. Monkeys were quarantined for 21 days before the study was conducted. At the end of this experiment, all of the 32 monkeys were deeply anesthetized by intravenous injection of pentobarbital sodium followed by exsanguination subsequently. And organs or tissue samples were obtained for histopathology.

### Experimental design

Animals were randomly divided into four groups: conventional rice (non-GM rice), positive control, 17.5% GM rice and 70% GM rice. Animals in the GM rice groups were fed on diets containing 17.5% or 70% GM rice for 182 days. During the quarantine and recovery periods, all animals were fed on diets containing 70% non-GM rice. During the treatment period, animals in the non-GM rice and positive control groups were fed on diets containing 70% non-GM rice for 182 days, and animals in the positive group were intravenously injected with cyclophosphamide (CP, Jiangsu Hengrui Medicine Co., Ltd. Jiangsu, China) every other day at dose of 20 mg/kg body weight for a total of 4 injections.

Throughout the study, animals were fed on 200 g/day of the special solid food. In addition to daily clinical observation and weekly body weight, T cell-dependent antibody response (TDAR) and spleen cell proliferation measurements, all parameters were measured during the 0, 1^st^, 3^rd^ and 6^th^ month and during the recovery period. TDAR was performed during the recovery period, and spleen cell proliferation was detected at animal dissection.

### Body weight, body temperature and electrocardiogram

Animals were observed twice daily to identify clinical abnormalities, mortality, and food consumption. Body weights were measured weekly through the study. Body temperature and electrocardiogram were examined twice in the quarantine period, 3 times in the study period and once in the recovery period.

### Hematology

The blood samples were collected from the saphenous vein in EDTA for hematology or sera separating gel to separate the sera for a chemistry analysis. The hematological parameters included the white blood cell (WBC) count, red blood cell (RBC) count, absolute and relative lymphocyte (LYMPH) count, absolute and relative neutrophil granulocyte (NEUT) count, absolute and relative eosinophilic granulocyte (EOS) count, absolute and relative basophilic granulocyte (BASO) count, absolute and relative monocyte (MONO) count, hemoglobin (HGB) level, hematocrit (HCT) level, mean corpuscular volume (MCV), mean corpuscular hemoglobin (MCH), mean corpuscular hemoglobin concentration (MCHC), platelet (PLT) count, mean platelet volume (MPV), and reticulocyte (RETIC) count (S1 Table). All hematology parameters in the blood samples were determined on an Advia 120 Hematology Analyzer (Bayer, Germany). In addition, blood smears were prepared for visual evaluation.

### Clinical chemistry

The following clinical chemistry parameters were measured using a Hitachi 7180 Automatic Biochemistry Analyzer (Hitachi, Japan): albumin (ALB), alkaline phosphatase (ALP), alanine aminotransferase (ALT), aspartate aminotransferase (AST), creatinine kinase (CK), lactate dehydrogenase (LDH), total bilirubin (TBIL), urea nitrogen (BUN), creatinine (CRE), glucose (GLU), cholesterol (CHO), triglyceride (TG), albumin/globulin ratio (A/G), total protein (TP), sodium (Na+), potassium (K^+^), chloride (Cl^-^), immunoglobulin (IgG and IgM) and complements (C3 and C4).

### Histopathology

The day after the last treatment, all of the 32 monkeys were deeply anaesthetized with pentobarbital sodium and subsequently killed by exsanguination. The organs, including the brain, pituitary gland, thyroid, parathyroid, submandibular gland, spinal marrow, thymus, sternum, heart, aorta, tongue, trachea, esophagus, lung, liver, gall bladder, kidney, spleen, adrenals, pancreas, stomach, duodenum, jejunum, ileum, cecum, colon, rectum, testis, epididymis, prostate, uterus, ovaries, vagina, breast, bladder, sciatic nerve, skeletal muscle, skin, eye, optic nerve, mesenteric lymph nodes and inguinal lymph nodes, were examined for histopathological changes, and the brain, pituitary, thyroid, thymus, heart, lung, liver, kidney, adrenals, spleen, testis, epididymis, prostate, ovaries and uterus were weighed.

All tissue samples were fixed in 4% neutral buffered formaldehyde (10% formalin), whereas the testes, epididymis and eyes were fixed in Davidson’s solution.

### Determination of immune parameters

#### Immunophenotyping of lymphocytes in the peripheral blood

Blood was collected in tubes containing heparin. After incubation with a T cell antibody mixture or B/NK cell antibody mixture for 20 min at room temperature in the dark, the red blood cells were removed using FACS lysing buffer. After two washes with phosphate buffered saline (PBS), the cells were fixed in PBS containing 4% paraformaldehyde. All samples were analyzed on a Flow Cytometer (FACS Calibur^**TM**^, BD, USA) using the CellQuest software. The following antibodies were used in immuno-phenotyping analysis: peridinin chlorophyll protein (PerCP)-conjugated mouse anti-human CD3 (T lymphocytes), fluorescein isothiocyanate (FITC)-conjugated mouse anti-human CD8 (T cytotoxic cells), R-phycoerythrin (PE)-conjugated mouse anti-human CD4 (T helper cells), PerCP-conjugated mouse anti-NHP CD45 (leukocytes), FITC-conjugated mouse anti-human CD20 (B cells), PE-conjugated mouse anti-human CD16 (NK cells). These antibodies and the lysing buffer used in the immuno-phenotyping were purchased from Becton, Dickenson and Company (Franklin Lakes, NJ, USA).

#### Determination of cytokines levels in serum

As for the clinical chemistry measurements, serum was collected from venous blood. Levels of Th1 cytokines (IFN-γ, IL-2, and TNF-β) and Th2 cytokines (IL-4, IL-5, and IL-6) were determined using a Cytometric Bead Array NHP Th1/Th2 Cytokine Kit (#557800, BD, USA) according to the manufacturer’s instructions. Briefly, after mixing with the 6 capture beads, standard or serum samples were incubated with PE detection reagent for 3 hours at room temperature in the dark. After being washed once with wash buffer, the samples were resuspended and analyzed by FACS using the CellQuest software in a manner similar to that used for the immuno-phenotyping of lymphocytes.

#### KLH-T cell dependent antibody response (KLH-TDAR)

The day after the last treatment, the monkeys were immunized with KLH (#H-7017, Sigma, Germany) via intravenous injection at a dose of 5 mg/kg b.w. Blood serum was collected 0, 4, 7, 11, 14, 21, and 28 days after the immunization. The levels of anti-KLH IgG and anti-KLH IgM were measured using monkey anti-KLH IgG ELISA kits and monkey anti-KLH IgM ELISA kits (#4010-4-INT; 4000-4-INT; Life Diagnostics, USA) according to the manufacturer’s instructions.

#### Mitogen-induced peripheral blood lymphocyte proliferation

Blood was collected in tubes containing heparin. Lymphocytes were isolated from the blood using a Ficoll-Hypaque solution and incubated with Lectin from phaseolus vulgaris (PHA-P, #L8754, Sigma, Germany). After incubation for 72 h, the number of lymphocytes was measured using a cell counting kit (CCK-8, Keygen, Nanjing, China). The lymphocyte proliferation index was calculated as described by Wessels SG et al [14].

#### Mitogen-induced splenocyte proliferation

Splenocyte suspensions were prepared as described by Song et al [15]. The spleen was weighed, and a part of the weighed spleen was aseptically transferred to cell culture dishes containing 2 mL of cold PBS. The spleen was cut into pieces and triturated through a 74 μm stainless steel mesh (CellCribble). The cell suspensions were then transferred to tubes, and red blood cells were removed using lysis buffer. After two washes with PBS containing 2% fetal calf serum (FBS), the cell suspensions were centrifuged at 100×g for 5 min at 4°C. The splenocytes were resuspended in 1 mL of RPMI 1640 culture medium (Gibco, Grand Island, NY, USA) containing 10% FBS. The number of splenocytes in each suspension was measured and adjusted to 2×10^**6**^ cells/mL. The proliferation of lymphocytes in response to PHA was evaluated in the same manner as peripheral blood lymphocyte proliferation.

### Statistical analysis

The data were statistically analyzed using SPSS 17.0, and the results are presented as the means±SD. The homogeneity of variance among groups and normality distribution were examined using Bartlett’s sphericity test. When Bartlett’s sphericity test was not significant, multiple groups were compared using a one-way ANOVA followed by Dunett’s post hoc comparisons tests. For non-normally distributed data, a non-parametrical test was performed using Kruskal–Wallis test followed by Dunett’s post hoc comparisons tests. *P*-values less than 0.05 were considered significant.

## Results

### Compositional analyses of rice and diets

The nutritional compositions of TT51-1, the parental rice (MingHui63) and conventional rice(non-GM rice) are shown in Table 1, while those of diets containing GM rice or non-GM rice are shown in Table 2. The crude protein, crude fat, crude fiber, crude ash, calcium and the phosphorus contents of TT51-1 were all essentially identical to those of the non-GM l rice. Diets containing GM rice did not differ from diets containing non-GM rice.

**Table 2:** Nutrient composition of diets containing non-GM rice or GM rice.

Contents	Non-GM rice	17.5% GM rice diets	70% GM rice diets
Bt protein (μg/g)	0.000±0.000	0.201±0.013	0.402±0.013
Crude protein (g/kg)	183.7±3.5	177.1±3.7	178.8±9.3
Crude fat (g/kg)	54.6±1.6	52.1±1.7	52.6±3.9
Crude fiber (g/kg)	9.5±0.2	13.1±6.4	17.9±12.8
Moisture (g/kg)	86.6±5.7	71.9±1.0	84.4±17.0
Crude ash (g/kg)	51.9±2.4	58.5±1.4	57.5±3.9
Calcium (g/kg)	10.7±0.3	11.4±0.4	11.3±0.3
Phosphorus (g/kg)	7.3±0.3	7.7±0.5	7.7±0.5

Note: Data are presented as the mean±SD.

### Effects of GM rice on animal appearance and food consumption

Food consumption was normal and all animals appeared normal, except for five animals that exhibited alopecia. Two of these animals were in the non-GM rice group.

### Effects of GM rice on body weight, body temperature and electrocardiogram

**Fig 1:**
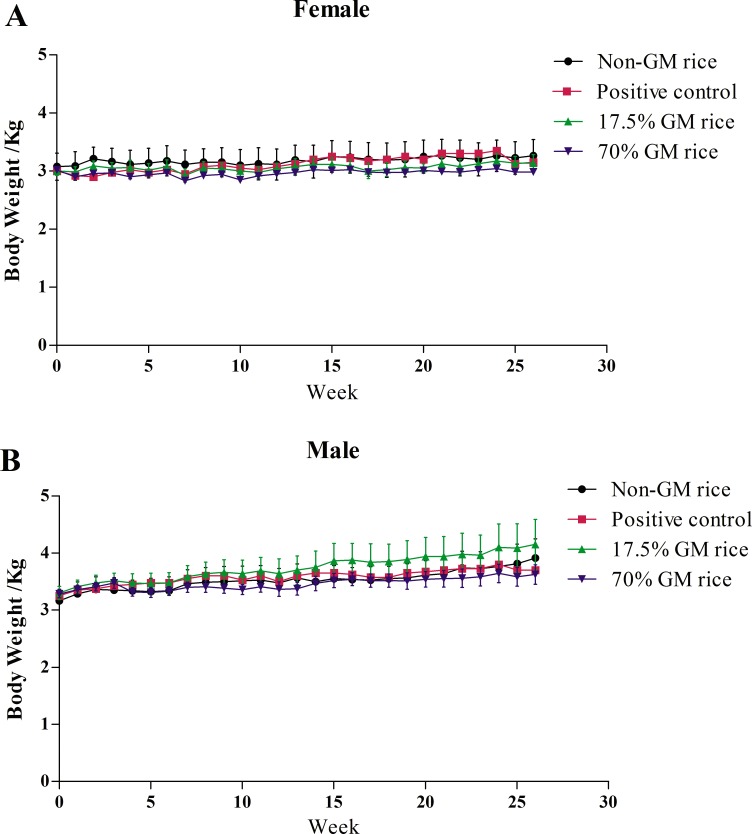
Body weights of monkeys fed on diets containing GM rice or non-GM rice. Body weights of female animals (A) and male animals(B) fed on diets containing genetically modified (GM rice) or non-GM rice increased steadily during the treatment period, and the body weights of animals in the GM rice groups and non-GM rice group did not significantly differ (*p*>0.05).

The body weights of female and male monkeys did not differ among any of the groups (Fig 1). The body temperatures of animals in the 70% GM rice group in the 1^st^ month and 3^rd^ month were significantly higher than those in the non-GM rice group (*p*<0.05) (Table 3). However, the changes were very small, and the body temperatures were within normal ranges.

**Table 3:** Body temperature of monkeys fed on diets containing GM rice or non-GM rice(°C).

Group	Pre-dose		Treatment				Recovery
	Week 1	Week 2	Month 1	Month 3	Month 6	Week 4	
**Non-GM rice**	39.00±0.40	38.63±0.51	38.89±0.45	38.84±0.56	38.93±0.47	38.65	
**Positive control**	39.33±0.43	38.30±0.35	39.08±0.30	39.10±0.18	39.15±0.26	/	
**17.5% GM rice**	39.19±0.39	38.70±0.46	39.29±0.60	39.19±0.45	39.28±0.72	39.45±0.44	
**70% GM rice**	39.24±0.43	38.90±0.62	39.59±0.33**	39.48±0.51*	39.43±0.53	39.13±0.56	

Note: Data are presented as the mean±SD. During the recovery period, there were 2 animals in the non-GM rice group and no animal in the positive control group.

*: Significantly different from the non-GM rice group at *p*<0.05.

**: Significantly different from the non-GM rice group at *p*<0.01.

The electrocardiogram data did not significantly differ among groups (Table 4).

**Table 4:** Electrocardiogram of monkeys fed on diets containing GM rice or non-GM rice.

Time	Group	Heart rate	P-R	Q-T	P	Q	R	S	ST	T
		bpm	ms	ms	mV	mV	mV	mV	mV	mV
**Pre-dose**	Non-GM rice	268±14	68±10	145±14	0.10±0.04	0.07±0.05	0.17±0.12	0.07±0.08	0.02±0.02	0.16±0.09
	Positive control	245±16	60±0	170±20	0.13±0.05	0.09±0.05	0.30±0.15	0.03±0.03	0.02±0.02.	0.09±0.03
	17.5% GM rice	272±18	65±9	153±15	0.13±0.04	0.08±0.06	0.34±0.22	0.04±0.04	0.04±0.03	0.14±0.08
	70% GM rice	260±14	63±12	157±12	0.10±0.04	0.09±0.09	0.30±0.17	0.05±0.05	0.03±0.03	0.17±0.07
**Treatment Month 1**	Non-GM rice	263±17	68±15	148±18	0.10±0.05	0.06±0.04	0.23±0.16	0.07±0.09	0.03±0.04	0.20±0.08
	Positive control	256±9	60±0	165±10	0.10±0.00	0.06±0.03	0.30±0.08	0.01±0.01	0.01±0.03	0.10±0.06
	17.5% GM rice	272±16	65±9	150±19	0.11±0.04	0.11±0.06	0.34±0.27	0.04±0.10	0.09±0.18	0.13±0.07
	70% GM rice	257±19	67±10	160±15	0.10±0.05	0.12±0.09	0.36±0.22	0.02±0.02	0.01±0.01	0.14±0.06
**Treatment Month 3**	Non-GM rice	255±14	68±10	153±10	0.09±0.02	0.05±0.03	0.22±0.14	0.06±0.05	0.01±0.02	0.17±0.09
	Positive control	240±13	70±12	155±38	0.10±0.00	0.09±0.03	0.36±0.21	0.01±0.00	0.03±0.05	0.08±0.06
	17.5% GM rice	258±23	65±9	150±21	0.11±0.02	0.10±0.07	0.42±0.28	0.03±0.02	0.04±0.04	0.12±0.08
	70% GM rice	251±19	73±10	160±12	0.09±0.04	0.09±0.06	0.33±0.21	0.04±0.04	0.02±0.02	0.14±0.06
**Treatment Month 6**	Non-GM rice	252±19	70±11	150±11	0.12±0.04	0.05±0.04	0.25±0.16	0.04±0.05	0.04±0.03	0.15±0.09
	Positive control	252±13	70±12	165±10	0.08±0.03	0.08±0.03	0.36±0.13	0.01±0.00	0.02±0.02	0.11±0.05
	17.5% GM rice	273±13	68±10	148±15	0.10±0.03	0.11±0.10	0.44±0.29	0.03±0.02	0.02±0.02	0.09±0.09
	70% GM rice	256±17	70±10	158±13	0.10±0.04	0.09±0.07	0.38±0.20	0.03±0.03	0.02±0.02	0.14±0.05
**Recovery**	Non-GM rice	245	70	170	0.10	0.03	0.11	0.15	0.05	0.23
	Positive control	/	/	/	/	/	/	/	/	/
	17.5% GM rice	280±17	65±10	150±12	0.11±0.02	0.12±0.13	0.49±0.35	0.04±0.04	0.07±0.04	0.11±0.05
	70% GM rice	268±22	65±10	160±16	0.09±0.03	0.08±0.05	0.38±0.29	0.02±0.02	0.01±0.03	0.15±0.09

Note: Data are presented as the mean±SD. During the quarantine period and the study period, there were 8, 4, 8 and 12 animals in the non-GM rice, positive control, 17.5% GM rice and 70% GM rice groups, respectively, whereas there were 2, 0, 4 and 4 animals in the four respective group during the recovery period.

### Effects of GM rice on hematology and clinical chemistry

After the injection of CP, the mean WBC and RETIC counts, the differential LYMPH and NEUT leukocyte counts, and the percentage of RETIC in the positive control group were significantly lower than those in the non-GM rice group, but the percentage of MONO was higher in the positive control group than that in the non-GM rice group (*p*<0.05) (Tables 5 and 6).

**Table 5:** Hematological data of monkeys fed on diets containing GM rice or non-GM rice.

Time	Group	WBC	%Neut	Neut	%Lymph	Lymph	%Mono	Mono	%Eos	Eos	%Baso	Baso
		(10^9^/L)	(%)	(10^9^/L)	(%)	(10^9^/L)	(%)	(10^9^/L)	(%)	(10^9^/L)	(%)	(10^9^/L)
**Pre-dose**	Non-GM rice	15.18±2.93	46.65±11.21	7.22±2.80	47.46±11.21	7.07±1.75	3.05±1.09	0.47±0.22	1.84±0.95	0.27±0.08	0.39±0.15	0.06±0.02
	Positive control	13.27±3.26	41.58±3.15	5.46±1.06	51.98±2.22	6.92±1.91	3.65±1.32	0.52±0.31	1.73±0.55	0.23±0.08	0.50±0.08	0.07±0.02
	17.5% GM rice	14.53±3.58	49.60±8.79	7.07±1.67	44.25±8.01	6.56±2.37	2.84±0.83	0.41±0.14	2.33±1.20	0.35±0.20	0.43±0.10	0.07±0.03
	70% GM rice	11.79±2.68	48.18±13.52	5.77±2.38	45.36±12.58	5.28±1.90	3.28±1.27	0.37±0.10	2.25±0.81	0.27±0.13	0.39±0.16	0.05±0.02
**Treatment Month 1**	Non-GM rice	13.67±3.30	42.93±13.35	5.85±2.50	50.84±13.83	6.98±2.82	3.13±1.16	0.42±0.16	1.68±0.79	0.22±0.09	0.89±0.40	0.13±0.07
	Positive control	14.59±3.73	36.33±9.26	5.30±1.75	57.45±8.43	8.38±2.29	3.25±0.79	0.49±0.23	1.55±0.58	0.23±0.12	0.78±0.57	0.11±0.06
	17.5% GM rice	13.22±3.79	39.18±16.86	4.89±2.03	54.53±16.56	7.48±3.73	3.18±0.77	0.41±0.12	1.90±059	0.26±0.14	0.63±0.28	0.09±0.06
	70% GM rice	13.07±3.72	46.16±14.11	6.31±3.14	48.02±13.03	6.04±1.50	2.70±0.93	0.33±0.07	1.98±1.36	0.25±0.17	0.55±0.31	0.07±0.03
**Treatment Month 3**	Non-GM rice	13.44±2.51	42.80±13.47	5.82±2.62	51.58±13.57	6.88±1.99	3.35±1.20	0.45±0.17	1.36±0.76	0.17±0.10	0.43±0.10	0.06±0.02
	Positive control	12.61±2.77	36.35±5.77	4.55±1.17	58.00±4.78	7.34±1.84	3.38±1.14	0.43±0.18	1.38±0.31	0.18±0.07	0.45±0.13	0.06±0.02
	17.5% GM rice	14.29±2.82	43.08±14.83	5.97±1.81	51.80±14.52	7.58±3.08	2.76±0.82	0.39±0.11	1.44±0.85	0.21±0.15	0.40±0.20	0.06±0.04
	70% GM rice	11.11±2.57	43.78±13.88	5.02±2.40	50.43±13.43	5.45±1.48	3.37±0.96	0.36±0.09	1.62±0.55	0.18±0.09	0.36±0.10	0.04±0.02
**Treatment Month 6**	Non-GM rice	12.78±1.49	43.28±10.63	5.50±1.35	51.25±11.00	6.59±1.79	3.39±1.02	0.43±0.13	1.33±0.94	0.16±0.10	0.36±0.05	0.03±0.01
	Positive control	5.41±0.69**	43.35±2.08	2.35±0.31*	44.95±5.73	2.44±0.46**	7.88±3.60**	0.43±0.19	1.98±1.32	0.10±0.06	0.18±0.10	0.01±0.01
	17.5% GM rice	14.74±3.37	43.99±12.42	6.32±2.01	50.98±13.11	7.69±3.35	2.84±0.87	0.41±0.13	1.05±0.59	0.15±0.11	0.29±0.16	0.05±0.03
	70% GM rice	12.12±3.13	46.22±11/35	5.68±2.51	47.78±10.85	5.72±1.60	4.03±1.11	0.48±0.12	0.93±0.56	0.12±0.08	0.27±0.09	0.03±0.01
**Recovery**	Non-GM rice	16.82	39.50	6.55	51.05	8.66	6.10	1.04	2.45	0.42	0.40	0.07
	Positive control	/	/	/	/	/	/	/	/	/	/	/
	17.5% GM rice	12.68±3.60	41.08±8.84	5.03±0.80	53.18±9.59	6.97±3.26	3.55±1.53	0.41±0.12**	1.38±0.47	0.18±0.08	0.30±0.00	0.04±0.01
	70% GM rice	12.05±2.45	44.58±20.81	5.66±3.86	48.80±19.67	5.59±1.92	4.38±0.46	0.53±0.13*	1.50±0.99	0.18±0.11	0.35±0.17	0.05±0.02

Note: Data are presented as the mean±SD. During the quarantine period and the study period, there were 8, 4, 8 and 12 animals in the non-GM rice, positive control, 17.5% GM rice and 70% GM rice groups, respectively, whereas there were 2, 0, 4 and 4 animals in the four respective group during the recovery period.

*: Significantly different from the non-GM rice group at *p*<0.05.

**: Significantly different from the non-GM rice group at *p*<0.01.

**Table 6:** Hematological data of monkeys fed on diets containing GM rice or non-GM rice.

Time	Group	RBC	HGB	HCT	MCV	MCH	MCHC	PLT	MPV	%Retic	#Retic
		(10^12^/L)	(g/L)	(%)	(fL)	(pg)	(g/L)	(109/L)	(fL)	(%)	(10^12^/L)
**Pre-dose**	Non-GM rice	5.67±0.66	135.13±20.08	47.28±6.29	83.39±5.13	23.80±1.65	285.75±14.38	406.00±76.87	9.46±1.09	1.60±0.66	87.63±30.87
	Positive control	6.11±0.69	146.50±11.68	49.70±4.45	81.55±4.05	24.05±1.34	295.25±7.63	370.50±16.05	9.60±1.78	1.40±0.67	81.25±33.33
	17.5% GM rice	5.82±0.64	138.00±18.80	47.86±6.86	82.09±5.49	23.71±1.68	288.63±8.11	398.88±90.18	9.24±0.82	1.54±0.41	88.50±18.34
	70% GM rice	5.98±0.77	141.58±18.14	49.35±5.42	82.78±5.02	23.68±1.24	286.08±8.94	372.83±101.11	9.33±1.21	1.33±0.33	80.00±19.92
**Treatment Month 1**	Non-GM rice	5.18±0.35	125.00±10.16	43.20±3.90	83.39±5.30	24.08±1.32	289.38±15.81	419.63±41.87	9.20±0.85	1.34±0.3	68.75±15.39
	Positive control	5.35±0.35	127.50±5.00	42.85±2.23	80.25±3.69	23.88±0.88	297.75±6.80	399.75±56.15	9.05±1.52	1.35±0.51	71.25±26.36
	17.5% GM rice	5.34±0.42	128.25±7.94	43.56±2.85	81.69±4.39	24.10±1.56	294.75±9.22	375.13±63.77	9.14±0.54	1.25±0.43	66.50±20.13
	70% GM rice	5.38±0.43	126.25±9.28	43.94±3.78	81.80±5.37	23.48±1.09	287.58±10.93	379.42±77.83	9.04±1.36	1.09±0.29	57.58±13.80
**Treatment Month 3**	Non-GM rice	5.19±0.44	124.13±8.31	42.60±2.85	82.28±4.61	23.98±1.18	291.88±13.41	445.13±93.09	9.11±0.77	1.64±0.80	81.75±34.62
	Positive control	5.55±0.38	129.75±5.32	43.23±1.97	78.05±3.78	23.45±0.61	300.25±7.50	405.75±46.64	9.10±1.44	1.28±0.59	69.50±28.45
	17.5% GM rice	5.32±0.38	124.25±11.06	42.15±3.14	79.33±4.76	23.38±1.68	294.75±8.84	425.50±78.18	9.01±0.59	1.23±0.27	58.25±22.79
	70% GM rice	5.33±0.50	124.00±10.30	42.89±3.69	80.75±4.83	23.32±1.33	289.08±7.67	397.33±84.31	9.14±1.20	1.38±0.52	71.67±24.51
**Treatment Month 6**	Non-GM rice	5.07±0.49	129.50±8.11	43.84±3.30	86.66±4.45	25.63±1.17	295.88±9.72	409.38±45.24	9.40±0.71	1.34±0.46	66.85±18.84
	Positive control	4.89±0.53	122.25±6.40	39.53±3.84	81.15±4.59	25.15±1.48	310.25±15.65	412.00±13.22	9.20±0.88	0.10±0.00**	4.38±1.71**
	17.5% GM rice	5.33±0.56	133.50±12.95	44.86±4.39	84.31±5.48	25.13±1.69	297.88±11.05	424.50±95.20	9.10±0.85	1.09±0.40	57.58±18.92
	70% GM rice	5.40±0.63	132.08±12.49	45.41±3.69	84.53±5.23	24.57±1.34	290/83±7.58	364.67±80.52	9.45±1.09	1.12±0.43	58.53±19.64
**Recovery**	Non-GM rice	4.55	123.00	39.90	87.65	26.95	307.50	343.00	9.45	1.10	49.50
	Positive control	/	/	/	/	/	/	/	/	/	/
	17.5% GM rice	4.89±0.45	129.75±8.18	40.98±2.03	84.15±6.13	26.65±2.55	316.50±8.89	434.00±87.96	8.68±0.62	1.25±0.49	59.53±18.82
	70% GM rice	5.48±0.87	137.50±11.09	44.73±3.36	82.50±7.18	25.38±2.47	307.50±7.42	327.00±81.18	8.65±1.01	1.03±0.56	52.73±23.10

Note: Data are presented as the mean±SD. During the quarantine period and the study period, there were 8, 4, 8 and 12 animals in the non-GM rice, positive control, 17.5% GM rice and 70% GM rice groups, respectively, whereas there were 2, 0, 4 and 4 animals in the four respective group during the recovery period.

*: Significantly different from the non-GM rice group at *p*<0.05.

**: Significantly different from the non-GM rice group at *p*<0.01.

In the 1^st^ month, the levels of ALT in the 70% GM rice groups were significantly higher than those in the non-GM rice group (*p*<0.05) (Table 7). In the 3^rd^ month, the levels of K^+^ in the GM rice groups and Cl^-^ in the 17.5% GM rice group were significantly lower than those in the non-GM rice group (*p*<0.05) (Table 8). Because the values of these changed parameters appeared to be within the normal ranges of values for monkeys of this age, these changes lacked toxicological significance.

**Table 7:** Clinical chemistry data of monkeys fed on diets containing GM rice or non-GM rice.

Time	Group	ALT	AST	ALP	CK	LDH	TBIL	BUN	CRE	GLU	CHO	TG
		(U/L)	(U/L)	(U/L)	(U/L)	(U/L)	(μmol/L)	(mmol/L)	(μmol/L)	(mmol/L)	(mmol/L)	(mmol/L)
**Pre-dose**	Non-GM rice	35.30±8.07	32.25±5.92	248.50±67.23	198.63±49.73	366.00±70.6	2.44±0.96	6.55±1.07	39.25±5.99	4.62±0.28	3.23±0.50	0.32±0.10
	Positive control	60.00±16.37**	39.50±4.65	298.25±29.57	217.75±68.68	425.75±58.28	2.60±0.36	6.40±0.24	36.25±5.12	4.68±0.34	3.77±0.85	0.26±0.09
	17.5% GM rice	36.63±15.71	38.38±7.80	237.88±93.43	234.25±85.91	414.25±83.97	2.78±0.25	6.93±1.04	37.75±3.49	3.87±0.56	3.39±0.60	0.36±0.13
	70% GM rice	48.42±9.89	40.08±11.02	262.00±90.38	202.92±46.43	483.42±148.95	2.66±0.67	6.78±1.32	40.33±6.62	4.61±0.96	3.56±0.69	0.32±0.10
**Treatment Month 1**	Non-GM rice	46.75±12.98	39.00±12.01	224.25±93.90	313.38±376.53	448.38±88.80	2.48±0.43	5.89±1.14	41.38±6.07	4.78±0.85	3.00±0.53	0.36±0.14
	Positive control	64.50±23.27	46.25±25.16	301.00±62.75	245.50±166.24	462.50±102.98	1.91±0.52	6.18±0.81	41.25±1.71	5.03±0.75	3.20±0.77	0.36±0.13
	17.5% GM rice	48.13±20.41	35.00±9.44	233.88±106.79	198.38±51.77	379.63±48.65	2.15±0.36	6.73±0.95	40.63±6.65	4.30±0.49	3.06±0.43	0.41±0.12
	70% GM rice	70.08±18.14*	42.42±14.12	233.08±71.20	318.25±301.80	438.00±118.19	2.50±0.71	6.34±0.97	45.17±6.24	4.75±1.15	3.45±0.65	0.46±0.25
**Treatment Month 3**	Non-GM rice	61.25±17.28	41.88±11.32	254.50±106.08	269.63±164.21	435.88±90.36	2.53±0.94	7.38±1.05	45.75±7.36	4.91±0.96	3.14±0.58	0.36±0.16
	Positive control	86.75±32.85	52.75±30.48	324.75±56.33	296.75±163.06	482.25±154.35	2.20±0.86	6.90±0.92	44.00±3.37	5.14±0.50	3.53±0.90	0.32±0.12
	17.5% GM rice	69.75±32.62	62.13±50.94	294.63±153.90	511.75±808.54	448.38±134.63	2.31±0.22	7.86±1.51	46.63±6.89	4.56±0.86	2.90±0.40	0.40±0.13
	70% GM rice	86.33±27.39	55.17±19.78	263.08±98.28	373.00±269.07	548.25±158.69	2.76±0.96	7.21±0.73	47.58±8.21	5.33±1.06	3.35±0.70	0.37±0.11
**Treatment Month 6**	Non-GM rice	42.63±12.05	40.63±12.36	295.38±128.35	220.50±63.03	439.13±101.23	3.93±0.58	6.73±0.59	34.75±6.36	4.87±0.78	2.78±0.46	0.37±0.12
	Positive control	64.50±20.82	43.25±8.42	416.00±113.57	172.75±32.64	398.25±21.19	3.98±0.51	5.53±0.64	35.50±7.55	4.28±0.45	3.33±0.41	0.25±0.09
	17.5% GM rice	50.13±24.90	56.25±34.11	347.25±173.86	298.88±181.97	447.88±125.31	4.15±0.60	7.20±1.34	36.13±8.08	4.45±0.61	2.89±0.62	0.41±0.13
	70% GM rice	56.25±13.66	49.42±19.91	334.08±142.35	240.25±98.57	532.58±126.99	4.19±0.88	6.30±0.90	37.58±7.05	4.88±0.81	3.30±0.68	0.36±0.10
**Recovery**	Non-GM rice	44.00	30.50	286.50	165.50	343.00	3.12	7.208	38.50	4.87	2.32	0.35
	Positive control	/	/	/	/	/	/	/	/	/	/	/
	17.5% GM rice	41.50±27.09	43.75±11.06	291.50±189.44	245.25±78.76	410.00±94.97	4.19±0.41	6.78±1.01	39.50±2.89	4.42±1.01	3.03±0.56	0.50±0.33
	70% GM rice	110.25±115.93	51.25±23.31	330.75±175.17	250.25±79.32	583.50±325.48	4.51±0.79	8.28±1.51	47.25±4.43	4.42±0.44	3.33±0.77	0.47±0.19

Note: Data are presented as the mean±SD. During the quarantine period and the study period, there were 8, 4, 8 and 12 animals in the non-GM rice, positive control, 17.5% GM rice and 70% GM rice groups, respectively, whereas there were 2, 0, 4 and 4 animals in the four respective group during the recovery period.

*: Significantly different from the non-GM rice group at *p*<0.05.

**: Significantly different from the non-GM rice group at *p*<0.01.

**Table 8:** Clinical chemistry data of monkeys fed on diets containing GM rice or non-GM rice.

Time	Group	TP	ALB	A/G	IgG	IgM	C3	C4	K+	Na+	Cl-
		(g/L)	(g/L)		g/L	g/L	g/L	g/L	(mmol/L)	(mmol/L)	(mmol/L)
**Pre-dose**	Non-GM rice	81.89±2.82	41.93±4.36	1.08±0.25	9.06±2.35	0.920.21	1.33±0.15	0.32±0.12	5.72±0.73	154.34±4.54	109.36±2.01
	Positive control	84.05±3.48	43.63±2.38	1.09±0.14	9.22±1.44	1.15±0.43	1.28±0.13	0.25±0.06	5.65±0.84	154.93±4.03	108.93±2.65
	17.5% GM rice	82.69±3.16	44.40±2.07	1.17±0.10	8.53±1.19	0.96±0.28	1.23±0.06	0.25±0.06	5.69±0.67	154.08±3.14	109.38±2.32
	70% GM rice	82.44±6.08	44.18±2.39	1.17±0.12	8.52±1.84	0.96±0.28	1.20±0.11*	0.23±0.09	5.68±0.64	153.36±3.27	108.18±2.23
**Treatment Month 1**	Non-GM rice	74.94±3.28	43.33±3.21	1.40±0.25	8.95±1.64	0.89±0.20	1.13±0.15	0.30±0.11	5.84±0.80	153.66±4.45	112.51±2.68
	Positive control	72.78±3.80	42.38±2.13	1.41±0.22	8.74±2.06	1.02±0.41	1.08±0.11	0.24±0.05	5.57±0.46	152.40±3.24	111.18±1.36
	17.5% GM rice	74.13±3.14	44.13±2.18	1.48±0.11	8.59±1.29	0.91±0.20	1.08±0.08	0.24±0.07	5.41±0.67	152.05±2.01	111.98±2.37
	70% GM rice	75.43±4.30	44.34±1.89	1.45±0.18	8.77±1.97	0.93±0.31	1.11±0.14	0.23±0.09	5.53±0.66	152.16±3.66	110.85±2.03
**Treatment Month 3**	Non-GM rice	76.70±4.85	42.94±3.83	1.32±0.30	10.59±2.60	1.16±0.22	1.28±0.16	0.39±0.20	6.31±0.72	153.05±4.36	112.61±2.07
	Positive control	77.18±5.23	44.15±2.65	1.36±0.24	10.88±2.92	1.41±0.53	1.24±0.13	0.30±0.09	6.03±0.43	151.75±1.92	111.38±1.03
	17.5% GM rice	77.23±4.98	45.46±2.27	1.45±0.16	9.83±1.55	1.12±0.31	1.21±0.13	0.30±0.10	5.22±0.49**	151.74±2.88	110.34±2.36
	70% GM rice	77.04±3.63	44.27±2.37	1.38±0.26	10.03±2.11	1.14±0.41	1.25±0.14	0.35±0.18	5.54±0.69*	151.98±3.17	109.52±2.04**
**Treatment Month 6**	Non-GM rice	79.31±3.31	41.41±2.43	1.10±0.09	7.24±1.54	0.50±0.11	0.91±0.11	0.21±0.06	5.32±0.43	151.04±3.05	106.59±1.78
	Positive control	79.85±6.95	42.45±1.84	1.19±0.33	7.43±2.30	0.62±0.27	0.90±0.12	0.19±0.05	5.17±0.22	150.35±1.83	107.30±2.43
	17.5% GM rice	83.10±4.27	44.21±2.81	1.15±0.14	7.42±1.35	0.55±0.22	0.87±0.07	0.19±0.05	5.00±0.42	151.23±2.05	105.74±2.15
	70% GM rice	81.53±5.48	42.83±2.41	1.13±0.19	7.23±2.03	0.54±0.24	0.92±0.12	0.20±0.08	5.08±0.44	151.22±2.82	105.72±1.34
**Recovery**	Non-GM rice	79.15	39.75	1.02	7.57	0.45	0.93	0.22	5.13	151.00	107.30
	Positive control	/	/	/	/	/	/	/	/	/	/
	17.5% GM rice	80.35±6.05	38.88±3.81	0.95±0.11	7.95±1.40	0.74±0.34	0.84±0.09	0.19±0.07	5.30±0.32	149.65±2.95	109.45±0.73
	70% GM rice	78.25±1.10	40.95±2.06	1.10±0.12	5.94±1.05	0.64±0.37	0.90±0.04	0.16±0.04	5.19±0.38	149.08±3.29	107.40±1.58

Note: Data are presented as the mean±SD. During the quarantine period and the study period, there were 8, 4, 8 and 12 animals in the non-GM rice, positive control, 17.5% GM rice and 70% GM rice groups, respectively, whereas there were 2, 0, 4 and 4 animals in the four respective group during the recovery period.

*: Significantly different from the non-GM rice group at *p*<0.05.

**: Significantly different from the non-GM rice group at *p*<0.01.

No other significant differences in hematology and clinical chemistry parameters were observed among any of the groups.

### Effects of GM rice on organ weights, macroscopic and microscopic evaluation

The absolute and relative weights of the thymus in the positive control group did not significantly differ from those in the non-GM rice group (Table 9).

**Table 9:** Organ weights of monkeys fed on diets containing GM rice or non-GM rice.

Organs	Non-GM rice (n = 4)^#^	Positive control (n = 4)^#^	17.5% GM rice (n = 4)	70% GM rice (n = 4)
**Treatment Termination**				
**Liver**				
Absolute weight(g)	74.60±10.32	59.18±12.78	63.05±7.98	55.90±6.03
Relative weight(g/100g)	1.89±0.21	1.77±0.34	1.88±0.19	1.69±0.16
**Kidney**				
Absolute weight(g)	12.30±1.25	12.25±1.07	11.05±0.71	12.58±0.61
Relative weight(g/100g)	0.31±0.04	0.37±0.05	0.33±0.04	0.38±0.04
**Spleen**				
Absolute weight(g)	3.620±0.861	3.237±0.588	4.529±1.297	3.856±1.192
Relative weight(g/100g)	0.092±0.024	0.096±0.012	0.136±0.040	0.115±0.025
**Thymus**				
Absolute weight(g)	1.835±0.386	0.990±0.244	2.199±0.972	1.869±1.021
Relative weight(g/100g)	0.047±0.011	0.029±.005	0.063±0.022	0.059±0.034
**Pituitary**				
Absolute weight(g)	0.061±0.007	0.058±0.011	0.057±0.013	0.065±0.020
Relative weight(g/100g)	0.002±0.000	0.002±0.001	0.002±0.000	0.002±0.000
**Thyroid**				
Absolute weight(g)	0.349±0.063	0.453±0.124	0.425±0.061	0.473±0.103
Relative weight(g/100g)	0.009±0.001	0.013±0.003	0.013±0.003	0.014±0.003
**Adrenals**				
Absolute weight(g)	0.467±0.052	0.465±0.113	0.478±0.125	0.506±0.104
Relative weight(g/100g)	0.012±0.003	0.014±0.003	0.015±0.006	0.015±0.004
**Recovery Termination**				
**Liver**				
Absolute weight(g)	55.2	/	66.73±14.28	56.00±8.30
Relative weight(g/100g)	1.42	/	1.59±0.08	1.41±0.15
**Kidney**				
Absolute weight(g)	10.25	/	12.30±1.75	10.95±1.55
Relative weight(g/100g)	0.26	/	0.30±0.04	0.28±0.04
**Spleen**				
Absolute weight(g)	2.717	/	3.751±1.022	3.067±0.938
Relative weight(g/100g)	0.071	/	0.093±0.038	0.079±0.030
**Thymus**				
Absolute weight(g)	1.885	/	1.547±0.641	1.652±0.948
Relative weight(g/100g)	0.05	/	0.037±0.015	0.043±0.028
**Pituitary**				
Absolute weight(g)	0.079	/	0.058±0.010	0.055±0.010
Relative weight(g/100g)	0.002	/	0.001±0.000	0.001±0.000
**Thyroid**				
Absolute weight(g)	0.466	/	0.460±0.093	0.274±0.061
Relative weight(g/100g)	0.012	/	0.011±0.002	0.007±0.001
**Adrenals**				
Absolute weight(g)	0.458	/	0.504±0.151	0.426±0.075
Relative weight(g/100g)	0.012	/	0.012±0.003	0.011±0.002

Note: Data are presented as the mean±SD.

^#^ During the recovery period, there were 2 animals in the non-GM rice group and no animals in the positive control group.

**Fig 2:**
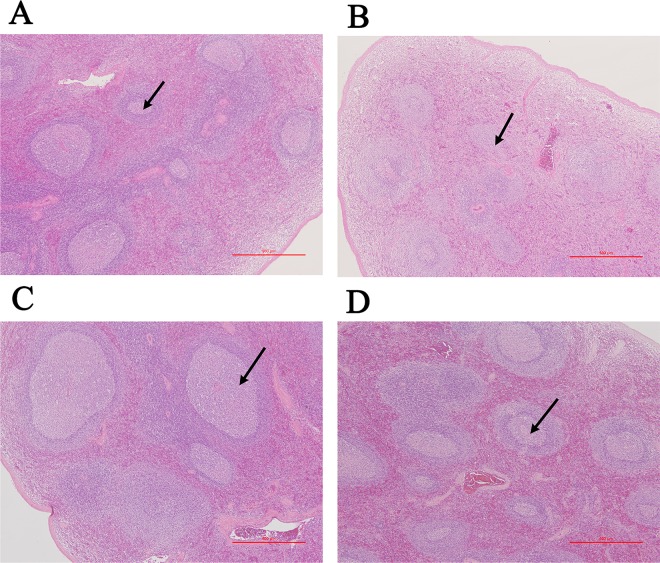
Microscopic evaluation of the spleens of monkeys fed on diets containing GM rice or non-GM rice. Microscopic evaluation of the spleen (40×) in monkeys in the non-GM rice group (A), positive control group (B), 17.5% genetically modified rice (GM rice) group (C), and 70% GM rice group (D). The spleens of monkeys in the positive group (B) showed atrophy and slightly fewer lymphocytes in the white pulp.

**Fig 3:**
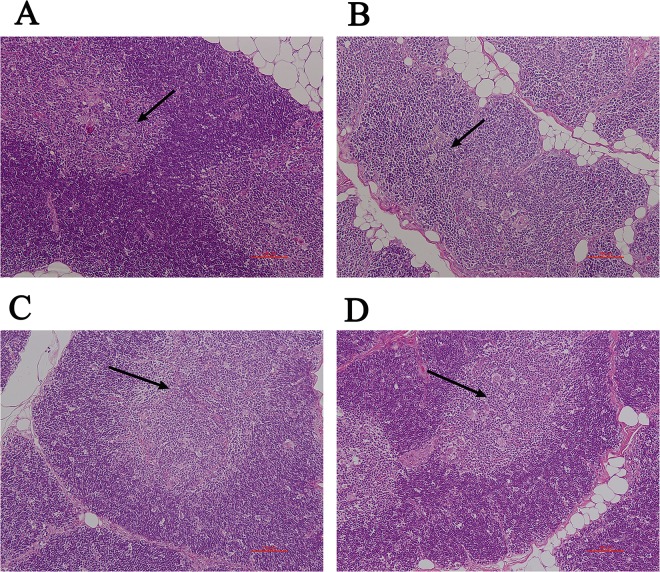
Microscopic evaluation of the thymus of monkeys fed on diets containing GM rice or non-GM rice (Stained with HE, 100×). Microscopic evaluation of the thymus (100×) in monkeys in the non-GM rice group (A), positive control group (B), 17.5% genetically modified rice (GM rice) group (C), 70% GM rice group (D). Thymus glands of monkeys in the positive group (B) show atrophy and moderately fewer lymphocytes in the cortex and medulla.

Fewer lymphocytes were observed in the white pulp of the spleen and the cortex and medulla of the thymus in the positive control group (Figs 2B and 3B). The macroscopic and microscopic evaluations did not reveal any other abnormalities in any of the groups.

### Immunotoxicity of GM rice

#### Effects of GM rice on immunophenotyping of lymphocytes in the peripheral blood

After the injection of CP, the proportion of CD3^**+**^CD8^**+**^ T lymphocytes significantly decreased, whereas the proportion of CD3^**+**^CD8^**+**^ T lymphocytes and the ratio of CD4^**+**^/CD8^**+**^ significantly increased compared with the respective parameters in the non-GM rice group (*p*<0.05) (Table 10). The immunophenotyping of lymphocytes in the peripheral blood of the GM rice groups and the non-GM rice group in response to treatment did not significantly differ.

**Table 10:** Immunophenotyping of lymphocytes in the peripheral blood of monkeys fed on diets containing GM rice or non-GM rice (%).

Group	Non-GM rice (n = 4)^#^	Positive control (n = 4)^#^	17.5% GM rice (n = 4)	70% GM rice (n = 4)
**Pre-dose**				
CD3+CD4+	60.85±4.07	56.48±4.93	58.98±9.40	55.29±6.38
CD3+CD8+	32.75±4.38	34.77±3.72	34.74±7.98	3.79±6.75
CD4+/CD8+	1.90±0.36	1.65±0.31	1.82±0.64	1.50±0.50
CD45+CD16+	19.17±6.15	17.34±5.82	19.30±7.11	19.55±5.22
CD45+CD20+	8.22±3.32	10.32±4.91	10.47±4.87	7.24±3.11
**Month 1**				
CD3+CD4+	63.53±6.45	62.68±4.40	57.81±10.54	58.54±9.09
CD3+CD8+	31.55±6.34	30.25±2.88	36.72±9.12	35.68±8.29
CD4+/CD8+	2.13±.66	2.10±0.34	1.74±0.80	1.76±0.58
CD45+CD16+	17.48±6.32	15.40±2.85	15.84±5.50	18.50±7.26
CD45+CD20+	7.42±4.00	8.50±3.82	7.96±3.62	5.58±2.48
**Month 3**				
CD3+CD4+	62.04±5.05	59.19±1.75	57.52±12.45	53.91±9.19
CD3+CD8+	31.87±4.50	32.35±2.56	36.55±10.41	39.00±9.02
CD4+/CD8+	2.00±0.45	1.84±0.20	1.79±0.86	1.48±0.50
CD45+CD16+	15.03±6.05	13.02±6.22	15.08±4.80	18.50±4.93
CD45+CD20+	7.57±2.44	8.98±2.40	8.52±2.54	7.82±2.51
**Month 6**				
CD3+CD4+	60.12±6.37	74.95±3.40*	54.22±13.57	52.77±9.18
CD3+CD8+	33.75±6.24	19.03±3.97*	39.73±12.91	39.84±9.14
CD4+/CD8+	1.86±0.52	4.08±0.92**	1.57±0.77	1.42±0.47
CD45+CD16+	15.69±4.77	18.96±11.34	15.40±4.47	18.40±6.09
CD45+CD20+	7.10±3.00	10.72±4.70	6.72±3.55	6.25±2.53
**Recovery period**				
CD3+CD4+	61.38	/	56.58±12.90	51.20±11.51
CD3+CD8+	30.34	/	37.67±12.02	42.28±12.81
CD4+/CD8+	2.03	/	1.70±0.85	1.34±0.59
CD45+CD16+	23	/	19.68±7.76	20.56±8.54
CD45+CD20+	6.25	/	6.45±2.23	5.81±2.95

Note: Data are presented as the mean±SD.

*: Significantly different from the non-GM rice group at *p*<0.05.

**: Significantly different from the non-GM rice group at *p*<0.01.

^#^During the recovery period, there were 2 animals in the non-GM rice group and no animal in the positive control group.

#### Effects of GM rice on serum levels of cytokines

During the 6^**th**^ month, the levels of IL-5, IL-4 and IL-2 of the 70% GM rice group were significantly lower than those of the non-GM rice group (*p*<0.05) (Table 11). However, the changed cytokines levels were within the normal ranges, and time-related and dose-related effects were not observed in the GM rice group. Thus, these changes were not attributed to GM rice treatment.

**Table 11:** Serum cytokine levels of monkeys fed on diets containing GM rice or non-GM rice(pg/mL).

Group	Non-GM rice (n = 4)^#^	Positive control (n = 4)^#^	17.5% GM rice (n = 4)	70% GM rice (n = 4)
**Pre-dose**				
IFN-γ	1.91±0.28	1.94±0.26	1.87±0.20	1.71±0.21
TNF	1.62±0.16	1.52±0.30	1.40±0.13	1.59±0.17
IL-6	1.93±1.37	1.42±0.22	1.36±0.29	1.50±0.27
IL-5	1.23±0.10	1.33±0.27	1.32±0.21	1.25±0.16
IL-4	1.23±0.10	1.28±0.11	1.22±0.06	1.21±0.11
IL-2	2.22±0.34	2.36±0.55	2.20±0.23	2.07±0.27
**Month 1**				
IFN-γ	1.75±0.13	2.02±0.52	1.87±0.35	1.70±0.24
TNF	1.47±0.17	1.79±0.49	1.62±0.27	1.46±0.22
IL-6	1.72±0.57	1.77±0.39	1.54±0.26	1.50±0.16
IL-5	1.18±0.09	1.43±0.48	1.31±0.17	1.18±0.11
IL-4	1.20±0.07	1.37±0.30	1.32±0.15	1.20±0.14
IL-2	2.09±0.31	2.52±1.02	2.46±0.60	2.02±0.49
**Month 3**				
IFN-γ	1.92±0.26	2.10±0.21	2.09±0.21	1.83±0.26
TNF	1.64±0.30	1.74±0.22	1.80±0.37	1.61±0.24
IL-6	1.74±0.41	1.87±0.39	1.85±0.31	1.72±0.23
IL-5	1.26±0.19	1.41±0.17	1.39±0.17	1.30±0.16
IL-4	1.33±0.18	1.46±0.16	1.49±0.26	1.37±0.19
IL-2	2.47±0.64	3.06±0.67	3.17±0.91	2.72±0.64
**Month 6**				
IFN-γ	2.14±0.19	2.10±0.28	1.91±0.23	1.96±0.21
TNF	1.93±0.16	1.82±0.16	1.76±0.25	1.67±0.24
IL-6	1.53±0.10	1.56±0.11	1.44±0.26	1.46±0.17
IL-5	1.43±0.07	1.4±0.06	1.36±0.16	1.29±0.12*
IL-4	1.56±0.06	1.52±0.12	1.43±0.18	1.36±0.15*
IL-2	3.38±0.29	3.27±0.37	2.91±0.62	2.64±0.61*
**Recovery period**				
IFN-γ	1.62	/	1.84±0.20	1.64±0.21
TNF	1.31	/	1.51±0.15	1.48±0.21
IL-6	1.66	/	1.41±0.07	1.56±0.38
IL-5	1.15	/	1.28±0.07	2.80±2.90
IL-4	1.17	/	1.25±0.05	1.28±0.10
IL-2	1.85	/	2.43±0.34	2.37±0.36

Note: Data are presented as the mean±SD.

*: Significantly different from the non-GM rice group at *p*<0.05.

**: Significantly different from the non-GM rice group at *p*<0.01.

^#^During the recovery period, there were 2 animals in the non-GM rice group and no animal in the positive control group.

#### Effects of GM rice on KLH-T cell-dependent antibody response

**Fig 4:**
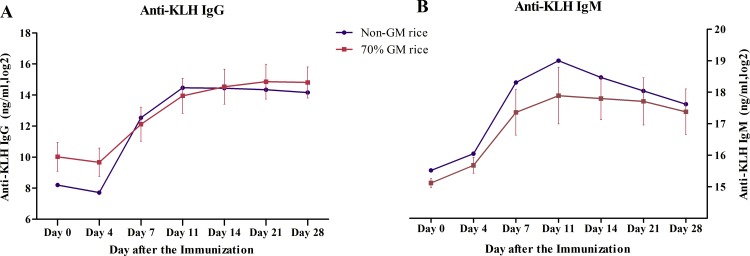
Serum levels of anti-KLH IgG and anti-KLH IgM after immunization. (A) Serum levels of anti-KLH IgG (A) in monkeys fed on diets containing genetically modified (GM rice) or non-GM rice reached a plateau on day 11 after the KLH-immunization. (B) Serum levels of anti-KLH IgM in monkeys fed on diets containing GM rice or non-GM rice were maximized 11 days after the KLH-immunization.

The serum levels of anti-KLH IgG and anti-KLH IgM did not significantly differ between the non-GM rice and 70% GM rice groups on day 0, 4, 7, 11, 14, 21, and 28 after immunization with KLH (*p*>0.05). Anti-KLH IgG reached into the platform stage on day 11, and anti-KLH IgM level reached the highest level on day 11 (Fig 4).

#### Effects of GM rice on mitogen-induced peripheral blood lymphocyte proliferation

No significant differences were observed among all groups in PHA-induced peripheral blood lymphocyte proliferation through the treatment (Table 12).

**Table 12:** Mitogen-induced peripheral blood lymphocyte proliferation in monkeys fed on diets containing GM rice or non-GM rice.

Group	Lymphocyte Proliferation Rate(%)			
	Non-GM rice (n = 4)^#^	Positive control (n = 4)^#^	17.5% GM rice (n = 4)	70% GM rice (n = 4)
**Pre-dose**	93.92±10.77	93.92±4.14	98.58±12.87	95.24±13.56
**Month 1**	1123.42±38.04	108.66±27.73	134.46±28.50	117.19±32.57
**Month 3**	128.98±28.98	153.09±40.30	191.56±91.69	154.36±57.32
**Moth 6**	110.34±42.79	73.68±17.56	141.23±44.12	123.99±42.60
**Recovery period**	139	/	157.20±51.40	160.87±42.34

Note: Data are presented as the mean±SD.

^#^ During the recovery period, there were 2 animals in the non-GM rice group and no animal in the positive control group.

#### Effects of GM rice on mitogen-induced splenocyte proliferation

The PHA-induced peripheral blood lymphocyte proliferation did not significantly differ among any of the groups at the termination of treatment and the termination of the recovery period (Table 13).

**Table 13:** Mitogen-induced splenocyte proliferation in monkeys fed on diets containing GM rice or non-GM rice (%).

Group	Non-GM rice (n = 4)^#^	Positive control (n = 4)^#^	17.5% GM rice (n = 4)	70% GM rice (n = 4)
**Treatment Termination**	208.22±49.68	173.85±39.92	198.04±56.20	214.80±38.36
**Recovery Termination**	281.67	/	182.67±64.64	178.97±76.30

Note: Data are presented as the mean±SD.

^#^ During the recovery period, there were 2 animals in the non-GM rice group and no animals in the positive control group.

## Discussion

The safety of GM foods has been in the public focus since the development of GM organisms. As an exogenous substance, Bt protein expressed in genetically modified rice may lead to allergic reactions or exert other effects on the immune system of human beings. This study was performed to evaluate the potential effects of the long-term feeding of rice containing Bt genes on the immune system of non-human primates.

The Cry1Ab/Ac gene was stably expressed in TT51-1 rice because the TT51-1 rice expressed 2.5μg/g Cry1Ab/Ac protein in our study, which is very similar to the levels reported by the Central China Agricultural University [16]. Cry1Ab/Ac protein may be thermally unstable because levels lower than the theoretical levels were detected in GM diets, which corroborates the results of previous studies [17].

The hematology and clinical chemistry are important parameters that affect general toxicity. They can reflect the effects of test materials on the function of organs, such as the liver and kidney. As conventional parameters in standard toxicity studies, they can provide early information on inflammation and may also provide information on the toxicity of substances to target organs. In our study, animals in the 70% GM rice group showed higher levels of ALT after treatment for 1 month, and animals in the GM rice groups showed lower levels of K^+^ and Cl^-^ than those in the non-GM rice group after treatment for 3 months. Previous studies showed dose-dependent decreases in the TG, CRE, ALB and GOT in female SD rats after the administration of a diet of GM rice containing the *cry1Ab* gene for 14 weeks [8]. In subchronic toxicity studies of Bt rice, changes in the TP, MCH, CRE or CHOL were observed in rats fed on GM rice containing *cry1Ab* [11][13], *cy1C* [12]^]^ or *cry2A* [18] for 90 days. Similar to the findings of our study, the values of the changed parameters were within normal ranges, and dose-dependence and related histological effects results were not found. Therefore, these changes in hematology and clinical chemistry were considered toxicologically meaningless.

The T-cell dependent antibody response is the immune function recommended for study by International Conference on Harmonization of Technical Requirements for Registration of Pharmaceuticals for Human Use (ICH) S8 guideline for additional immunotoxicity studies following standard toxicity studies [19]. It provides an overall measurement of host immune function by evaluating various aspects of immune responses, including antigen processing and presentation, B and T lymphocyte interactions, antibody production, and cytokine-dependent isotype class switches. Thus, it is a sensitive immune function assay to detect changes in immune function prior to hematology and lymphoid tissue histopathology evaluation [20,21]. This study is the first report of the T-cell dependent antibody response in an immuno-toxicological evaluation of Bt rice. According to our results, after 6 months of treatment, the levels of anti-KLH IgG and anti-KLH IgM in the GM rice group and the non-GM rice group showed similar trends, and these findings consistent with those of previous studies [14][22]. Immunophenotyping is also recommended by ICH S8 as a method for additional immunotoxicity studies and is increasingly used to evaluate the safety of new drugs. Changes in the specific lymphocyte populations in the peripheral blood are a useful indicator of changes in humoral and cellular immunity. However, the immuno-phenotyping of animals in the GM Bt rice group in our study did not reveal changes.

Regarding the immunotoxicological parameters detected in this study, only the serum levels of cytokines (IL-2, IL-4, and IL-5) significantly differed between animals in the 70% GM rice group and the non-GM rice group. Cytokine changes may indicate humoral immunity damage. However, the differences were very small, and the cytokine levels of monkeys in the 70% GM rice group were within normal ranges for monkeys of that age. Moreover, the proportion of Th cells (CD3+CD4+ lymphocytes) and T-cell dependent antibody response did not change. Therefore, the changes in the serum cytokine levels were not attributed to toxicity. Longer-term feeding studies will be required to evaluate the effects of GM rice intake, especially the effects on the aforementioned changed parameters, including the clinical chemistry parameters.

In general, GM Bt rice treatment did not affect the immunotoxicological parameters in the present study. Similar to our findings, Song et al [14] and Kroghsbo et al [9] reported that mice or rats fed on Bt crops expressing Cry1Ah or Cry1Ab protein for 30 or 90 days did not exhibit adverse immunotoxicological effects. Although changes in the immune response have previously been observed in fish and mice following Bt maize consumption [23,24], the inconsistency in the results between studies are likely due to the use of different animal models[15]. Finally, we concluded that monkeys fed on a diet of GM rice containing the Cry1Ab/Ac gene for 6 months did not exhibit adverse immunotoxicological effects.

### Conclusion

This 182-day feeding study showed that feeding cynomolgus monkeys on a diet of genetically modified rice containing the *cry1Ab/Ac* gene (TT51-1) did significantly affect clinical parameters, except for some of the clinical chemistry parameters, the body temperature and the serum cytokine levels. However, these differences were not considered to be biologically significant. Thus, the consecutive administration of GM rice containing the Cry1Ab/Ac gene for 6 months did not exert adverse immunotoxicological effects under the conditions of this study.

## Supporting Information

S1 Table Abbreviations and Explanations. (DOC)
