# Supplementary material for: Immunotoxicological Evaluation of Genetically Modified Rice Expressing Cry1Ab/Ac Protein (TT51-1) by a 6-Month Feeding Study on Cynomolgus Monkeys
Source: PLoS One. 2016 Sep 29;11(9):e0163879. doi: 10.1371/journal.pone.0163879 (PMC5042482; doi:10.1371/journal.pone.0163879)
Supplement: S1 Table — (DOC) [file pone.0163879.s001.doc]

**S1 Table. Abbreviations and Explanations**

| **No.** | **Abbreviations** | **Explanations** | **No.** | **Abbreviations** | **Explanations** |
| --- | --- | --- | --- | --- | --- |
| 1 | A/G | albumin/globulin ratio | 29 | INF-γ | interferon-gamma |
| 2 | ALB | albumin | 30 | K^+^ | potassium |
| 3 | ALP | alkaline phosphatase | 31 | KLH | keyhole limpet hemocyanin |
| 4 | ALT | alanine aminotransferase | 32 | LDH | lactate dehydrogenase |
| 5 | AST | aspartate aminotransferase | 33 | LYMPH | lymphocyte |
| 6 | BASO | basophilic granulocyte | 34 | MCH | mean corpuscular hemoglobin |
| 7 | Bt | *Bacillus thuringiensis* | 35 | MCHC | mean corpuscular hemoglobin concentration |
| 8 | BUN | urea nitrogen | 36 | MCV | mean corpuscular volume |
| 9 | C3 | complement 3 | 37 | MONO | monocyte |
| 10 | C4 | complement 4 | 38 | MPV | mean platelet volume |
| 11 | CCK-8 | cell counting kit-8 | 39 | Na^+^ | sodium |
| 12 | CHO | cholesterol | 40 | NEUT | neutrophile granulocyte |
| 13 | CK | creatinine kinase | 41 | NHP | non-human primate |
| 14 | Cl^-^ | chloride | 42 | PBS | phosphate buffered saline |
| 15 | CP | cyclophosphamide | 43 | PE | R-phycoerythrin |
| 16 | CRE | creatinine | 44 | PerCP | peridinin-chlorophyll protein complex |
| 17 | ELISA | enzyme-linked immuno sorbent assay | 45 | PHA | Lectin from phaseolus vulgaris |
| 18 | EOS | eosinophilic granulocyte | 46 | PLT | platelet |
| 19 | FBS | fetal calf serum | 47 | RBC | red blood cell |
| 20 | FITC | fluorescein isothiocyanate | 48 | RETIC | reticulocyte |
| 21 | GLU | glucose | 49 | TBIL | total bilirubin |
| 22 | GM rice | genetically modified rice | 50 | TDAR | T cell-dependent antibody response |
| 23 | GOT | glutamic oxaloacetic transaminase | 51 | TG | triglyceride |
| 24 | HCT | hematocrit | 52 | Th1 | T helper type 1 cell |
| 25 | HGB | hemoglobin | 53 | Th2 | T helper type 2 cell |
| 26 | IgG | immunoglobulin G | 54 | TNF | tumor necrosis factor |
| 27 | IgM | immunoglobulin M | 55 | TP | total protein |
| 28 | IL | interleukin | 56 | WBC | white blood cell |
